# Genetic variants in the G gamma-globin promoter modulate fetal hemoglobin expression in the Colombian population

**DOI:** 10.1590/1678-4685-GMB-2019-0076

**Published:** 2020-04-22

**Authors:** Cristian Fong, Yesica Mendoza, Guillermo Barreto

**Affiliations:** 1 GIOD Group, Faculty of Dentistry, Universidad Cooperativa de Colombia, Pasto, Nariño, Colombia; 2 Human Molecular Genetics Group, Biology Department, Universidad del Valle, Cali, Valle del Cauca, Colombia.

**Keywords:** Sickle cell anemia, fetal hemoglobin, gamma globin, regulation of gene expression, Colombia

## Abstract

Fetal hemoglobin (HbF) is a determining factor for the development of sickle cell anemia. High HbF levels lower the intensity of symptoms of this disease. HbF levels can vary in patients with sickle cell anemia and individuals without the disease. The purpose of this study was to identify the genetic variants in the G gamma-globin gene promoter that can modulate HbF expression in patients with sickle cell anemia and healthy individuals from Colombia. In total, 413 bp of the G gamma-globin gene promoter were sequenced in 60 patients with sickle cell anemia and 113 healthy individuals. The allelic and genotype frequencies of the identified variants were compared between individuals with low and high HbF for both patients and healthy individuals. In total, we identified 15 variants in both groups, only three of which were shared between patients and healthy individuals. In healthy individuals, sites -16 and -309 (rs112479156) exhibited differences in allele frequencies. The mutant allele of -16 lowered the production of HbF, whereas the mutant allele of -309 increased its production. These results reveal the presence of different mechanisms of HbF regulation between patients with sickle cell and healthy individuals.

The expression of fetal hemoglobin (HbF), a type of hemoglobin, starts around the fifth week of gestation. HbF consists of two types of globin chains: alpha and gamma. HbF synthesis stops during the perinatal period, at which time it is replaced by adult hemoglobin (Bank, 2006). In adults, HbF normally comprises less than 0.6% of the total hemoglobin content, and its production is limited to a specific group of cells called F cells. However, HbF levels among adult individuals can vary by more than 10-fold this amount (Rochette *et al*., 1994).

HbF is extremely important for the management of hemoglobinopathies because it has been reported that high HbF levels decrease the clinical severity of these diseases (Powars *et al*., 1984). HbF expression is under substantial genetic control with 89% heritability (Garner *et al*., 2000). Three quantitative trait loci (QTL) that intervene in HbF regulation have been identified. These sites are located on chromosome 2 in *BCL11A* (Uda *et al*., 2008), on chromosome 6 in the intergenic region between *HBS1L* and *MYB* (Menzel *et al*., 2007), and on the G gamma-globin promoter in a polymorphism located at position -158 (Gilman and Huisman, 1984). However, these three QTLs do not explain the total variability of HbF expression (Liu *et al*., 2016), which means that unidentified variants can influence the synthesis of HbF.

Sickle cell anemia is a disease caused by the presence of an adult hemoglobin variant known as hemoglobin S (HbS) (Pauling *et al*., 1949). Low oxygen tension polymerizes HbS, resulting in the deformation and destruction of red blood cells, which causes an inflammatory response in endothelial tissue. This state induces the adhesion of blood cells to endothelium, initiating vaso-occlusive events that induce severe pain and loss of organ function (Hebbel *et al*., 2004). The presence of HbF has been associated with a decrease in HbS polymerization and therefore less destruction of red blood cells and symptoms associated with the disease (Steinberg *et al*., 1997).

Promoters of globin genes have binding sequences to regulatory agents that can influence HbF synthesis (Traxler *et al*., 2016; Liu *et al*., 2017; Martyn *et al*., 2017). This investigation focused on the identification of genetic variants in the nuclear region of the G gamma-globin gene (HBG2) promoter and the determination of the effect of these variants on HbF synthesis in patients with sickle cell anemia and healthy individuals. Our results identified two genetic variants that can influence HbF synthesis in the Colombian population without sickle cell disease.

A total of 1136 blood samples (4 mL in Vacutainer® tubes containing ethylenediaminetetraacetic acid) were randomly collected from healthy individuals and patients with sickle cell anemia inhabiting the Pacific and Atlantic coasts of Colombia. Of these, 39 were from the San Jerónimo Hospital and ABO Laboratories in Montería (Atlantic coast), 184 were from the Louis Pasteur Laboratory and the Casa del Niño Hospital (Napoleón Franco) in Cartagena (Atlantic coast), 820 were from Buenaventura (Pacific coast), and 93 were from the Valle University Hospital and the Noel Club Children’s Clinic Foundation in Santiago de Cali (Pacific coast). The participants were individuals aged > two years who had not undergone a blood transfusion in the last three months and who had not been treated with hydroxyurea or any other drug that could increase HbF levels. The Ethics Committee of Universidad del Valle approved this research project (Approval Act 09-09). The participants or their legal representatives provided signed informed consent before their entry to the survey. Samples were stored at 4 °C in the Genetics Section of the Human Molecular Genetics Laboratory at the Universidad del Valle, Meléndez campus. From the 1136 samples, 60 individuals were diagnosed with sickle cell anemia (SS group), and a random sub-sample (n = 113) of individuals without sickle cell anemia was created (AA group).

DNA was extracted from each sample using the salting-out method (Miller *et al.,* 1988). DNA was then quantified via spectrophotometry (NanoDrop 2000, Thermo Fisher, USA), and working dilutions were prepared with a final concentration of 50 ng/µL. HbF was quantified using the alkaline denaturation technique for hemoglobin. HbF content was measured via spectrophotometry at 450 nm (Singer *et al*., 1951). This procedure was performed in the hematology laboratory of Valle University Hospital. SS individuals who had HbF levels exceeding 10% were included in the high HbF subgroup. This limit has been defined as the minimum HbF value for avoiding significant organ damage (Powars *et al*., 1984). HbF levels greater than 2% were considered high for the AA group (Rochette *et al*., 1994).

The genotype in the HbS locus was determined via PCR amplification of a 596-bp fragment of the beta-globin gene and subsequently digested with the *Ddel* restriction enzyme (Attila *et al*., 2004). The digestion reaction was performed according to the manufacturer’s instructions. For this, 10 µL of the amplified sample, a final concentration of 1 x reaction buffer, and 0.5 U/µL *Ddel* (Promega, USA) were mixed to achieve a final volume of 25 µL. Incubation was performed in a warm water bath at 37 °C for at least 16 h.

The primers CAGGCCTCACTGGAGCTACAGAC (forward) and ACCTCAGACGTTCCAGAAGCGAGT (reverse) were used to amplify the promoter region of *HBG2.* This sentence should be: The premix was defined as follows: buffer 1X; , 0.98 µM; dNTPs, 0.01 µM; primers, 0.12 µM; *Taq* DNA polymerase, 1 IU; and DNA, 50 ng. The amplification program was initiated with heating at 92 °C for 5 min, followed by 30 cycles of denaturation at 93 °C for 36 s, annealing at 70 °C for 1 min, and extension at 72 °C for 1.5 min. Subsequently, a final extension was performed at 72 °C for 3 min. Confirmation of the amplification was performed via electrophoresis using a 29:1 polyacrylamide gel at 8%, with a 413-bp band corresponding to the promoter region. The determination of the nucleotide sequence of *HBG2* promoter region (413 bp) was performed using Sanger-type sequencing with the BigDye® Terminator v3.1 Cycle Sequencing kit (Applied Biosystems, USA) following the amplification program suggested for this kit. Sequencing was performed using an ABI 3130 unit (Applied Biosystems) that was available at the Human Molecular Genetics Laboratory at the Universidad del Valle.

The sequences were processed and analyzed using Sequencing analysis v. 5.3.1 (Applied Biosystems) and aligned with the SeqScape program v. 2.7 (Applied Biosystems) where the different polymorphisms were identified. The allelic frequency of each single nucleotide polymorphism (SNP) was determined via direct counting in both the AA and SS groups. An estimation of the gametic phases was performed using the Arlequin program v. 3.5 (Excoffier and Lischer, 2010) and the Hardy–Weinberg (H–W) equilibrium for each locus separately. We used the package Haplo.stats for the R environment to determine the haplotypes association with HbF (http://CRAN.R-project.org/package=haplo.stats).

The allelic frequencies of the SNPs were compared in individuals with high and low HbF for both the SS and AA groups using the Raymond & Rousset test (Raymond and Rousset, 1995) with Arlequin v. 3.5 software. The sites that exhibited significant differences were corroborated via analysis of variance (ANOVA) using Statistica software v. 7.0 (Hilbe, 2007). Linkage disequilibrium (LD) was evaluated using Haploview software v. 4.2 (Barrett *et al*., 2005).

Within the 413 bp analyzed for the nuclear promoter region of *HBG2,* we found 15 variants in the entire population sample. The variants were observed at positions -356, -325, -324, -322, -317, -309, -307, -271, -203, -168, -161, -158, -152, -53, and -16. Six of these variants had previously been reported in NCBI: -324 (rs78485026), -317 (rs74929542), -309 (rs112479156), -307 (rs112219751), -271 (rs113622787), and -158 (rs7482144). However, no reports were found for the remaining nine sites. Seven variants were identified in patients with sickle cell anemia, and 11 were observed in healthy individuals. It is important to note that only three of these polymorphisms (-309, -307, and -158) were shared between patients and individuals without the disease (Tables 1 and 2).

**Table 1:** Allele frequency of the polymorphisms observed in the promoter region of the G-gamma globin gene in patients with sickle cell anemia. Column H—W (p value) shows the p-value from the Hardy-Weinberg equilibrium test. The Raymond & Rousset (p value) column shows the p-value from the test. Seven polymorphisms were identified. There are no significant differences in the allele frequency of these sites between patients with high and low HbF levels.

		SS (HbF < 10%) (n=46)		SS (HbF < 10%) (n=14)		
Locus	Alleles	Frequency	H–W (p value)	Frequency	H–W (p value)	Raymond & Rousset (p value)
-356	A	0.10	1.00	0.18	1.00	0.31
	G	0.90		0.82		
-324	G	0.09	1.00	0.14	1.00	0.47
	A	0.91		0.86		
-317	T	0.10	1.00	0.14	1.00	0.50
	C	0.90		0.86		
-309	T	0.89	0.36	0.89	1.00	1.00
	C	0.11		0.11		
-307	T	0.87	1.00	0.86	1.00	1.00
	C	0.13		0.14		
-271	G	0.07	1.00	0.11	1.00	0.68
	A	0.93		0.89		
-158	G	0.83	0.00	0.93	0.04	0.33
	A	0.17		0.07		

**Table 2:** Allele frequency of the polymorphisms observed in the promoter region of the G-gamma globin gene in individuals without sickle cell anemia. Column H–W (p value) shows the p-value from the Hardy-Weinberg equilibrium test. The Raymond & Rousset (p value) column shows the p-value from the test. 11 polymorphisms were observed. Only sites -309 and -16 presented differences in the allelic frequency between individuals with high and low HbF.

		AA (HbF < 2%) (n=40)		AA (HbF > 2%) (n=73)		
Locus	Alleles	Frequency	H–W (p value)	Frequency	H–W (p value)	Raymond & Rousset (p value)
-325	C	0.99	1.00	1.00	NA	0.44
	T	0.01		0.00		
-322	C	0.99	1.00	1.00	NA	0.44
	T	0.01		0.00		
-309	T	0.98	1.00	0.85	0.02	0.00
	C	0.02		0.15		
-307	T	0.96	0.06	0.98	1.00	0.70
	C	0.04		0.02		
-203	C	1.00	NA^a^	0.99	1.00	1.00
	G	0.00		0.01		
-168	C	0.99	1.00	1.00	NA	0.44
	T	0.01		0.00		
-161	A	0.02	0.01	0.00	NA	0.19
	G	0.98		1.00		
-158	G	0.85	0.06	0.83	0.00	0.72
	A	0.15		0.17		
-152	C	0.99	1.00	1.00	NA	0.44
	T	0.01		0.00		
-53	C	1.00	NA	0.99	1.00	1.00
	T	0.00		0.01		
-16	C	0.91	0.33	0.98	1.00	0.04
	G	0.09		0.02		

a NA= not applicable.

Table 1 shows the allelic frequencies in these polymorphic sites in the SS group for patients with high and low HbF levels. All polymorphisms excluding that located at -158 displayed H–W equilibrium. Similarly, we did not observe significant differences in the allelic frequencies between the two groups. Within the AA group, the vast majority of polymorphic sites presented H–W equilibrium excluding the -309 and -158 sites in the high HbF subgroup and -161 in the low HbF subgroup (Table 2). Raymond and Rousset’s analysis revealed differences in the allelic frequencies at the -309 and -16 sites between individuals with high and low HbF.

ANOVA was performed to corroborate the differences in allelic frequencies observed in the AA group. Therefore, the normality assumptions (p-value = 0.157) and variance homogeneity (p-value = 0.998) of HbF levels were evaluated. ANOVA revealed that individuals homozygous for TT at -309 had lower HbF levels than those with at least one mutated C allele (TC and CC) (p-value = 0.014). For the polymorphism at position -16, individuals with the CC genotype had higher HbF levels than those with the CG or GG genotype (p-value = 0.018) (Figure 1). We identified 14 and 12 haplotypes in the AA and SS groups, respectively. Only the haplotype 5’-CCCCGCCTAGGCTCG-3’ exhibited an association with HbF in the AA group.

**Figure 1:**
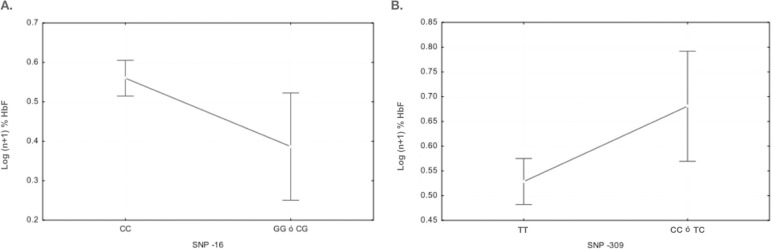
The effects of-16 and-309 onfetalhemoglobin synthesis. (A) The change fromCto Gat-16 causes a decrease inthe synthesis of fetalhemoglobin in individuals without sickle cell anemia. (B) The change from TtoC causes an increase in the synthesis of fetal hemoglobin in individuals without the disease.

Regarding LD between the restriction sites, sites -356, -325, -317, -309, -307, and -271 formed a linkage block with high levels of LD in the SS group. Site -158 exhibited intermediate levels of LD. Concerning healthy individuals, a linkage block was formed between sites -325 and -161. However, unlike the case of SS patients, this block has intermediate LD levels between some sites. LD levels were intermediate outside the linkage block, and some sites did not display LD, which could indicate recombination events (Figure 2).

**Figure 2:**
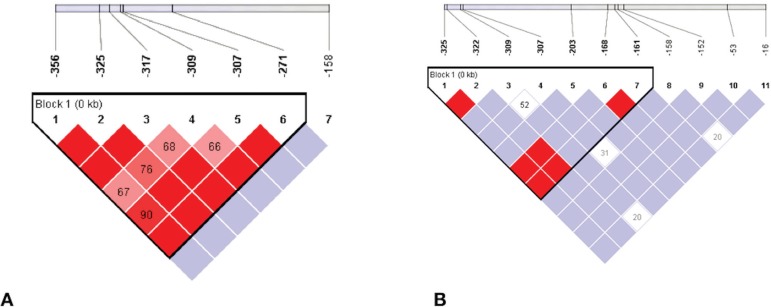
The degree of imbalance between variants in the G gamma globin gene promoter. The graph shows the result of the Haploview program. The red boxes denote a high logarithm of odds for linkage (LOD), whereas the violet squares denote a low LOD. The white squares denote a LOD of 0. The bar at the top represents the relative position of each of the variants. Panel A shows a high degree of LD between variants, generating a linkage block from -356 to -271 inindividualswith sickle cell anemia. Panel B showsthe degree ofLD amongvariants observedinindividualswithoutthe disease.Alinkage block was formed from -325 to -161. A lower LD can also be observed between the sites in comparison with those in patients with sickle cell disease.

HbF is a factor that modulates the severity of sickle cell anemia, and hence, its regulation has been well studied (Basak and Sankaran, 2016; Dulmovits *et al*., 2016; Liu *et al*., 2016). Genetic variants (SNPs) have been associated with HbF expression (Li *et al*., 2006; Thein and Menzel, 2009). In our study, we observed different groups of genetic variants between individuals with sickle cell anemia and healthy individuals, which could be the product of differences in the ethnic component between affected and unaffected individuals. Patients with sickle cell anemia have a high percentage of African heritage [>70% (Fong *et al*., 2013)], whereas individuals without the disease come from a mixed population with a predominance of European heritage followed by Amerindian ancestry (Ossa *et al*., 2016).

Only two polymorphisms (-309 and -16) displayed differences in allelic frequencies between individuals with high and low HbF levels, although this difference was only observed in individuals without sickle cell anemia. Previous reports of these two variations are scarce. For both cases, noninterference with HbF expression has been reported. In the case of -16, this polymorphism alters a putative CACCC box located between the TATA box and transcription start site. Despite this, -16 did not affect HbF expression in the adult or fetal state in an African American population (de Vooght *et al*., 2007). In our results, individuals with at least one mutant allele at -16 had lower HbF levels than those with the wild-type genotype. This result is consistent with the previous finding in cell studies in which mutations between the TATA box and “cap site” decrease promoter function (Amrolia *et al*., 1995). Our results do not necessarily mean that -16 is a determinant of the regulation of HbF expression. One possible explanation is that -16 is linked to other polymorphism that modulates HbF expression in the study population, but this was not evaluated in the present study. The different result between the present study and previous research could be the product of population differences, such as the presence or absence of miscegenation or other historical factors that can generate differences in the relationship between loci in different populations. These factors would explain the inability to replicate the findings of association studies performed in extremely different populations. Furthermore, -16 was not observed in individuals with sickle cell anemia, and this may mean that this variant is linked to the normal allele or that its presence (or the presence of the causal polymorphism) may represent extremely adverse effects in the affected individuals.

In the case of -309, this variant has been identified in patients with sickle cell anemia in Brazil, in which the mutant allele is widespread. However, as observed for -16 in African Americans, -309 was not associated with HbF levels (Barbosa *et al*., 2010). In our AA group, individuals with at least one mutant allele (G) had higher HbF levels than those with the wild-type genotype. Differences in the degree of association of genetic variants with HbF have been previously found. Pule *et al*. found no association between 18 SNPs and HbF in patients with sickle cell from Cameroon, although these same SNPs were associated with HbF in a population from Tanzania and Sardinia (Pule *et al*., 2016). These results illustrate that HbF regulation can have different mechanisms and that these differences can vary among populations (Solovieff *et al*., 2010). However, it is worthy of note that the sample size is a limiting factor in our study, and it could be necessary to increase the sample size to verify these results.

A noteworthy observation is that although site -158 has been associated with high HbF levels in patients with sickle cell anemia, in our population, no difference was found in allelic frequency between patients with high and low HbF levels. This observation may mean that -158 is not a functional site for the regulation of HbF but it is linked to the authentic regulatory factor. Akinsheye *et al*. propose that the SNP rs10128556 has significant effects on HbF expression and that this may be linked to the SNP rs7482144 (-158) (Akinsheye *et al*., 2011).

Two variants found at low frequencies in individuals without sickle cell anemia, -53 and -203, are located adjacent to two polymorphisms (-50 and -202) that generate a binding site for stage selector proteins that cause hereditary persistence of HbF (Jane *et al*., 1992). Sites -53 and -203 may generate a similar effect because they were observed in two individuals in the high HbF group in individuals without the disease, but this observation must be verified.

Chromosomes A and S also displayed differences in the LD pattern. Individuals with sickle cell disease had a higher degree of LD at sites excluding -158. Contrary to this, there was a weak or ambiguous link between the variants in the AA group. This result may be caused by differences in heterozygosity between both chromosomes in this region. The S chromosomes displayed heterozygosity of 0.37, and A chromosomes exhibited heterozygosity of 0.76 (result not shown). These differences in heterozygosity could be the product of an interbreeding process that is more widespread in healthy populations than sickle cell populations.

In conclusion, chromosomes S and A contain different groups of genetic variants in the *HBG2* promoter. No variants that were associated with HbF expression in patients with sickle cell anemia were found in our population, but two variants (-16 and -309) are associated to the HbF regulatory process in individuals without sickle cell disease. The presence of different groups of polymorphisms could also indicate that there may different mechanisms of HbF regulation between patients with sickle cell anemia and individuals without the disease.
